# Updating the editorial policies and practices of the Journal of the Brazilian College of Surgeons: a matter of survival

**DOI:** 10.1590/0100-6991e-20233695EDIT01-en

**Published:** 2023-11-23

**Authors:** Gerson Alves Pereira, Andreia Feitosa do Carmo, Julia Castro Neves, Ramiro Colleoni

**Affiliations:** 1 - FOB/USP, Curso de Medicina de Bauru - Bauru - SP - Brasil; 2 - Universidade Federal de São Paulo, Biblioteca do Campus São Paulo - São Paulo - SP - Brasil; 3 - Colégio Brasileiro de Cirurgiões, Departamento de Publicações - Rio de Janeiro - RJ - Brasil; 4 - Universidade Federal de Sao Paulo, Escola Paulista de Medicina - São Paulo - SP - Brasil

## EDITORIAL

The Journal of the Brazilian College of Surgeons (JBCS) has a very rich and diverse background. Launched in 1930 as the “Bulletin of the Brazilian College of Surgeons” it became gradually a traditional and relevant medical journal, although it was published with varying periodicity. The current name was changed in 1967 by decision of the National Board of the BCS and since 1974 it was published bimonthly on a regular basis. A single continuous annual online volume was adopted since January 2020.

In these almost 50 years of continuous publication, the JBCS has gained in importance and scope, currently being indexed in significant databases such as SciELO, LILACS, SCOPUS, SJR and PUBMED, increasing the availability of published articles and contributing to the advancement of national scientific knowledge.

In the last two years, we have updated our editorial policies and practices to maintain international indexing. Recently, the digital preservation of all the papers published since 2009 in JBCS was achieved. Soon, more than 1200 full text articles will be available in PubMed Central - PMC (https://www.ncbi.nlm.nih.gov/pmc/journals/4491/).

Currently only 48 of the 98 Brazilian health science journals indexed in SciELO have their articles available in full text in Medline/PMC.Our efforts will now be directed towards obtaining indexation in the Web of Science, in order to have an impact factor, which will enhance the visibility of the JBCS, both nationally and internationally. We use CiteScore that is the number of citations received by a journal in one year to documents published in the three previous years, divided by the number of documents indexed in Scopus published in those same three years. Our CiteScore in 2015 was 0.5 (2012 to 2015), in 2019 it was 1.0 (2016 to 2019) and in 2023 it reached 1.8 (2019 to 2022). There is much more room for growth, but it doesn’t just depend on internal factors within the journal and the Brazilian College of Surgeons.

In this two-year period, our initial efforts were to analyze all the manuscripts that were under review and establish a new proposal for submitting and reviewing articles. There was a marked improvement in the timing of all the journal’s processes, from receipt of the manuscript, document analysis (1- adequacy of the number of words in the whole text and in each section of the article (automatic), 2- CEP approval document, 3- Term of absence of conflicts of interest, 4- Term of authorship, and 5- Equator Checklist), checking of content and admissibility by the editor, carrying out the similarity test (Ithenticate); The manuscript is then sent to the reviewers for evaluation, the evaluation reports are sent to the authors with a decision as to whether or not to publish it and, once approved, the paper is sent for layout, translation into English, payment of fees, approval by the author and publication on the JBCS website and on the PMC portal.

None of this would have been possible without the valuable participation of our reviewers from the most varied areas of surgery, who have worked hard to meet the necessary deadlines so that JBCS can further improve its speed indicators for evaluating manuscripts.

The quality of articles published in JBCS has been assured by the rigorous peer review process ensuring that only good quality articles are published, following international standards that support the guidelines and recommendations of the International Committee of Medical Editors (ICMJE), Committee on Publication Ethics (COPE), the Council of Science Editors (CSE) and the World Association of Medical Editors (WAME).

Our editorial policy seeks to value the manuscripts received, passing through the content analysis and admissibility criteria, so that it can, at least, receive a first opinion on the quality of the article submitted. Some problems in the evaluated manuscripts have drawn attention: 1) low quality of the scientific text, especially the method, 2) lack of following instructions to authors that are updated on the website (https://www.revistadocbc.org.br/instrucoes -to-authors), 3) similarity test with high percentages due to the lack of adequacy of bibliographic references throughout the text, being returned for corrections and 4) difficulties for authors in the review process in making the suggested adjustments in a study with inadequate design. An example of this are submitted integrative and systematic reviews that rarely follow the standards defined in the instructions to authors.

For each new step in this climb towards improving the quality of articles and visibility of Brazilian scientific publications to maintain and achieve new international indexations, the number of criteria that need to be met increases, both in terms of the journal’s internal processes, quality and agility of reviewers, but also in the academic and scientific quality of the articles submitted. Therefore, we have to adopt individual and institutional measures that will ensure the highest quality in national scientific publications, including the training of human resources (at undergraduate, medical residency and postgraduate levels), training of advisors, public and private incentive policies scientific projects, maintenance of active and functional research groups in different areas of surgery in all regions of the country, and also the qualification of reviewers.

Among the years JBCS become an important and relevant Brazilian journal in the field of Surgery, publishing papers in diverse areas of surgical research as Head and Neck Surgery, Acute Care Surgery, Digestive Surgery, Experimental Surgery, General Surgery, Minimally Invasive Surgery, Surgical Oncology, Pediatric Surgery, Thoracic Surgery, Vascular Surgery, Colorectal Surgery, Medical Education, Digestive Endoscopy, Healthcare Management (quality improvement, patient safety and health network), and Transplant.Other surgical specialties that are not listed above may submit articles of interest within selected topics as Emergency, Trauma, Oncology, Medical Education and Healthcare Management). Articles from several other health science fields have been refused since they fall outside this scope and unfortunately they will not find good local journals for publication.

Over the next few years, several innovations are being evaluated to be help our team to move forward with the mission to disseminate high-quality research in Surgery. In order to accomplish this mission, the Brazilian College of Surgeons should coordinate with the various surgical research groups and collaborate to expand opportunities for surgical research, particularly multicenter studies.
